# Can ureteric stone impaction be predicted preoperatively using noninvasive parameters? A prospective study

**DOI:** 10.1080/20905998.2025.2495498

**Published:** 2025-04-21

**Authors:** Mohamed Samir, Mohamed Rafik Elhalaby, Amr Moustafa Nasef, Ahmed Higazy, Mohamed Ismail

**Affiliations:** a Urology, Ain Shams University Hospitals, Cairo, Egypt; b Urology, National Institute of Urology and Nephrology, Cairo, Egypt

**Keywords:** UWT, Stone impaction, Ureteroscope, NCCT, ESR, CRP

## Abstract

**Objective:**

To determine the potential predictive value of different non-contrast computed tomography (NCCT) parameters and acute phase markers (ESR and CRP) for identifying impacted ureteric stones preoperatively.

**Methods:**

We included 70 patients with ureteric stones who underwent ureteroscope (URS) in this study. The NCCT parameters and acute phase reactants were measured and compared in the impacted and non-impacted stone groups using univariate and multivariate analysis.

**Results:**

Out of the 70 patients, 35 had impacted ureteric stones. Using univariate analysis, we found that stone volume, stone location, hydronephrosis grade, and ureteral wall thickness (UWT) were the variables affecting the impaction, while by multivariate analysis, no significant association was detected between impaction and stone volume (*p* = 0.42). No association was found between the acute phase reactants and prediction of stone impaction Based on the receiver operating characteristic (ROC) curve analysis, UWT cutoff of 1.9 mm is an excellent predictor of impaction with 72.2% sensitivity, 94.1% specificity, and an overall accuracy level of 82.9%.

**Conclusion:**

Stone location, hydronephrosis grade and UWT were the best predictors for ureteric stone impaction preoperatively.

## Introduction

Impacted ureteric stones represent a common health problem causing many challenges in their proper management [1]. They are defined as stones remaining in the same position for more than 2 months duration [2] and another one is the inability of the contrast material or guidewire to pass beyond [3].

Stone impaction results in ureteral wall inflammation complicated by oedema, polyps, and fibrosis [4]. Moreover, it was linked to a higher incidence of urosepsis [5]. Also, it is related to many ureteroscopy (URS) associated complications and decreases its stone free rate [6,7].

Considering this information, it is important to predict stone impaction. However, many investigators have tried to reveal the value of numerous parameters with variable conclusions. Examples involve ureteral density in non-contrast computed tomography (NCCT) [8], ureteral wall thickness (UWT), C-reactive protein (CRP), erythrocyte sedimentation rate (ESR) [2], grade of hydronephrosis [9], age, stone position [10], and the ratio of CT attenuation value of the ureter above to below stones [11].

We aimed in our study to determine the potential predictive value of different NCCT parameters and acute phase markers (ESR and CRP) for identifying impacted ureteric stones preoperatively.

## Patients and methods

### Sample size

Using the PASS 15 program for sample size calculation, reviewing results from a previous study by Ozbir et al. showed that the stone free rate among the impacted stone group was 85%, also, the impaction stone formula (ISF) is the most precise preoperative predictor of impacted stones in patients with ureteral stones and area under the curve (AUC) = 0.958 based on these findings and after 20% adjustment for dropout rate a sample size of at least 30 patients achieves 99% power to detect a difference of 0.45 between the AUC under the null hypothesis of 0.5000 and an AUC under the alternative hypothesis of 0.9500 using a two-sided z-test at a significance level of 0.05. The data are continuous responses. The AUC is computed between false positive rates of 0 and 1. The ratio of the SD of the responses in the negative group to the standard deviation of the responses in the positive group is 1 [12].

### Study setting

This prospective study was conducted from 1 March to 31 August 2024. Ethical approval of our hospital was obtained (no. MS91/2024), and informed consent to participate was signed by the patients before sharing. Patients aged 18 years or more with single ureteric calculus for URS after failed medical expulsive therapy (MET) with silodosin 8 mg daily for 1 month were included. Exclusion criteria included patients with previous stone passage, prior ureteral intervention, known inflammatory condition, on anti-inflammatory drugs, active urinary tract infection (UTI), renal impairment, urinary tract congenital anomalies or vesicoureteric stones.

Eighty-one patients were eligible to be included in the study complying with the inclusion criteria and of whom 11 patients were excluded for different reasons. The assessment for each patient was adequate medical history, physical examination, and laboratory investigations (urine analysis, culture, creatinine, ESR, and CRP). Radiologic investigations involved NCCT done for the diagnosis and pelviabdominal ultrasound with KUB (kidney, ureter, and bladder X-ray) (NCCT if needed) done after 1 month of MET.

An expert radiologist blinded to the impaction status and the outcome using General Electric Optima 660 128 slice CT scanner measured the following NCCT parameters: stone site, density using Hounsfield Unit (HU), volume, hydronephrosis grade, perinephric fat stranding, UWT, HU of the ureter below the stone, HU of the ureter above the stone, and ratio of HU below/above the stone.

The serum level of CRP was measured using nephelometry (Atellica, Siemens). Concentration of CRP above 1 mg/L was considered positive. Also, ESR was measured at room temperature in an upright vertical position using standardized Westergren tube for one hour. It was considered positive if its value was >15 mm/hr in males and >20 mm/hr in females. Stone volume was measured in mm^3^ = 0.167 × π x width x length x height. The UWT was assessed using the default software at x 6 magnification of the axial view, measuring the soft tissue between the stone and the periureteric fat, not involving any fat stranding. Our study's primary endpoint was to identify the variables affecting stone impaction.

### Surgical procedure

All patients were operated upon by the same team of expert endourologists. Lower ureteric stones were managed by semi-rigid ureteroscope (43 mm Karl Storz 6.5–9.5 fr) and disintegrated using pneumatic lithotripsy (EMS Swiss Lithoclast 2) by a 0.8 mm metal probe. While, upper and mid-ureteric stones were managed either by semi-rigid or flexible ureteroscopy (Lithovue, Boston scientific 7.7–9.5fr) with or without an access sheath (13fr) according to the surgeon’s preference and intraoperative scenario and disintegrated using Holmium:YAG laser (Quanta System Litho) by a 365 µm laser fiber. Stone dusting was done using laser setting of 0.5 J energy & 20 hz frequency. Stone fragmentation was done using 1.5 J energy & 5 hZ frequency. A stent was applied in all cases.

**Figure 1.**
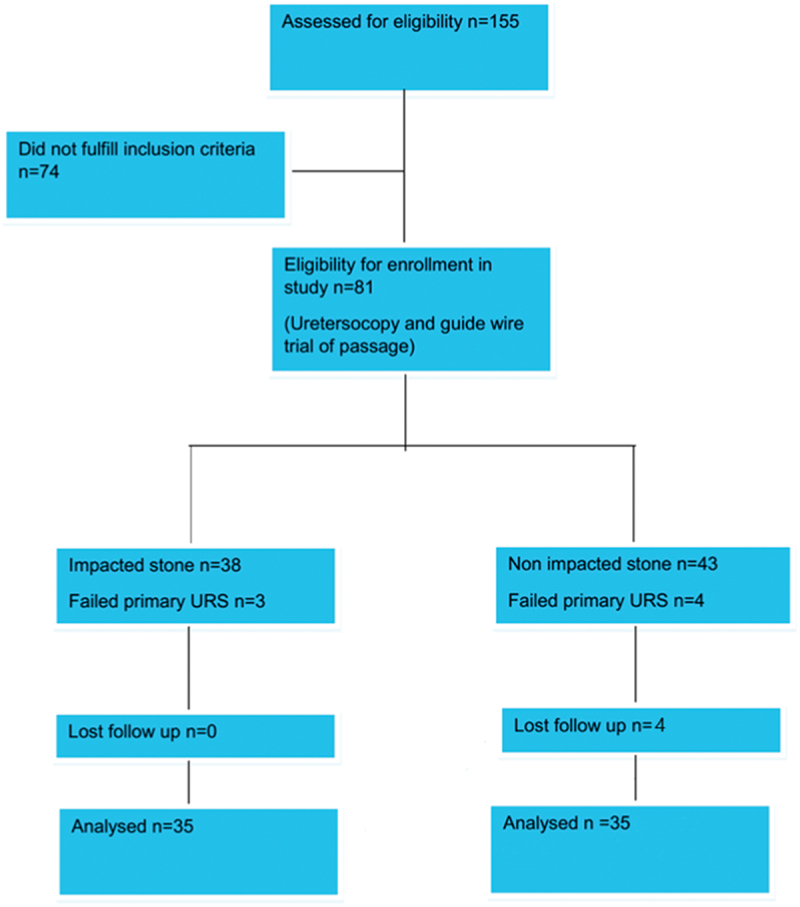
Study flow-chart.

Stone impaction was judged by the initial attempt to pass a guidewire (0.035”) (Coloplast) to the renal pelvis under fluoroscopic guidance and confirmed by URS thereafter. Therefore, patients were divided into 2 groups impacted and non-impacted (Figure 1).

### Statistical analysis

Normality of distribution was investigated using the Shapiro-Wilk test of distribution normality. Categorical data was shown in counts and frequencies (%) with associations between them being investigated using the Chi Square test of independence. Quantitative parameters were described as mean and standard deviation (SD) or as median and inter-quartile range (IQR), depending on the distribution of each variable. Comparison between groups regarding study parameters was made using 2 sample student’s t-test. Non-parametric alternatives were utilized when indicated. Multivariate logistic regression analysis was conducted to identify the predictors of stone impaction among those that were statistically significant after univariate analysis.

## Results

There was no evidence of association between stone impaction and gender (*p* = 0.821) or age (*p* = 0.398). We found that the stone site was significantly associated with impaction status (*p* < 0.001). Sixty-six percent of impacted ureteric stones were in the upper ureter, 20% were mid-ureteric and 14% were in the lower ureter. Stone volume was also statistically significant, as it was larger in the impacted with a median of 0.3 cm^3^ compared to the non-impacted group with a median volume of 0.2 cm^3^ (*p* = 0.003). Stone density was not statistically significant (*p* = 0.53) with a mean of 827.9 hU in the impacted and 790 hU in the non-impacted group.

There was no significant relation between perinephric fat stranding and stone impaction (*p* = 0.99). Both hydronephrosis and UWT were significantly associated with stone impaction (*p* < 0.001). However, HU of ureter above stone (p = 0.12), HU of ureter below stone (*p* = 0.61), and the ratio of HU of ureter below to above the stone (*p* = 0.072) did not show any statistically significant difference between the 2 groups Table 1.

**Table 1.** Patients’ demographics and perioperative characteristics.

	Stone impaction		
	Yes (*n* = 35) N (%)	No (*n* = 35) N (%)	*p*-value
**Gender**			
Female Male	15 (42.9)	18 (51.4)	0.821
	20 (57.1)	17 (48.6)	
**Age (years) Mean ± SD**	44.1 ± 11.9	41.8 ± 10.9	0.3986
**Stone site**			
Lower	5 (14)	15 (42.8)	**< 0.001**
Mid	7 (20)	18 (51.3)	
Upper	23 (66)	2 (5.9)	
**Stone volume (cm3)** Med (IQR)	0.3 (0.2–0.53)	0.2 (0.105–0.34)	**0.003**
**Stone (HU)** Mean ± SD	827.9 ± 250	790 ± 257.7	0.5312
**Hydronephrosis**			**< 0.001**
None	0 (0)	3 (8.5)	
Mild	9 (25.7)	24 (68.5)	
Moderate	14 (40)	7 (20)	
Marked	12 (34.3)	1 (2.9)	
**Perinephric fat stranding**			0.99
Yes	3 (8.5)	2 (5.8)	
No	32 (91.5)	33 (94.2)	
**UWT (mm)** Med (IQR)	2.3 (1.9–2.8)	1.5 (1.25–1.65)	**< 0.001**
**HU of ureter above stone** Med (IQR)	7 (3–12)	4 (0.9–9.5)	0.1218
**HU of ureter below stone** Mean ± SD	23.8 ± 9.42	22.3 ± 10.8	0.6155
**Ratio HU below/above** Med (IQR)	2.95 (2–4.95)	3.67 (2–6)	0.0726

HU: Hounsfield Unit, UWT: ureteral wall thickness.

The CRP values were positive in 4 patients in each group. Also, ESR was positive in only 1 patient in the non-impacted and 2 patients in the impacted group. Both inflammatory markers were statistically insignificant (*p* = 0.99).

Logistic regression was done. After involving the 4 significant parameters found in the univariate analysis, we found that stone location, hydronephrosis grade and UWT were the independent variables affecting the impaction. Stones with upper ureteric location (*p* = 0.044), marked hydronephrosis (*p* = 0.048) and higher UWT (*p* = 0.011) are more prone to be impacted. In contrast, no significant association was detected between impaction and stone volume (*p* = 0.42) Table 2.

**Table 2.** Logistic regression to identify predictors for impaction.

	OR (95% CI)	*p*-value
Stone location (Upper)	8.634 (1.2 to 90.45)	**0.0441**
Hydronephrosis (Marked)	24.585 (1.45 to 1)	**0.0487**
Stone size	4.412 (1.1 to 167.12)	0.4245
UWT	13.418 (2.17 to 131.09)	**0.0110**

UWT: ureteral wall thickness.

**Figure 2.**
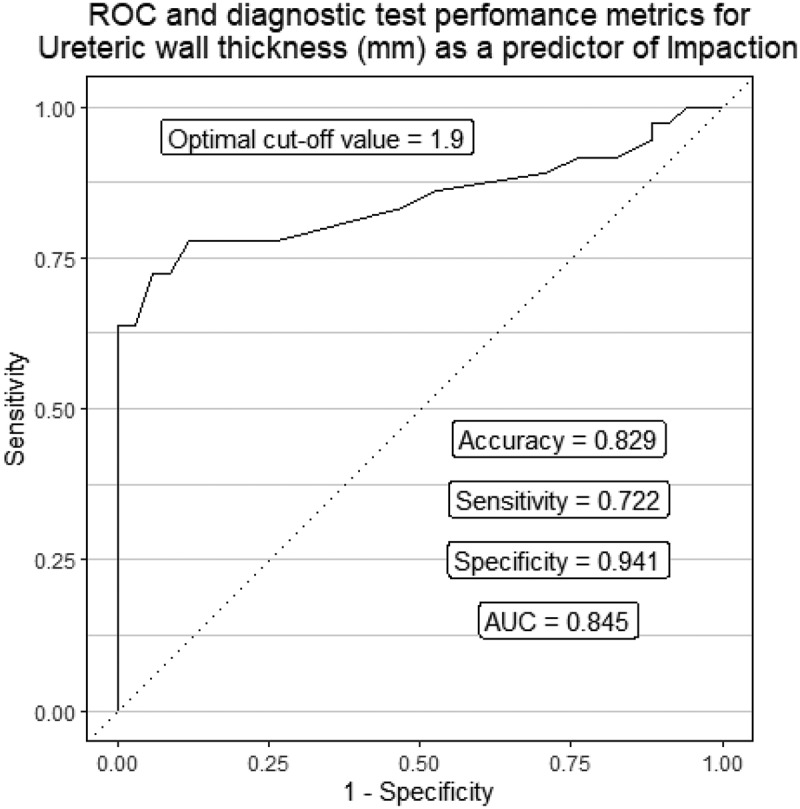
Diagnostic test performance of ureteric wall thickness as a predictor of impaction.

Based on the receiver operating characteristic (ROC) curve analysis, UWT cutoff value of 1.9 mm is an excellent predictor of impaction with 72.2% sensitivity, 94.1% specificity, and an overall accuracy level of 82.9% (Figure 2).

**Figure 3.**
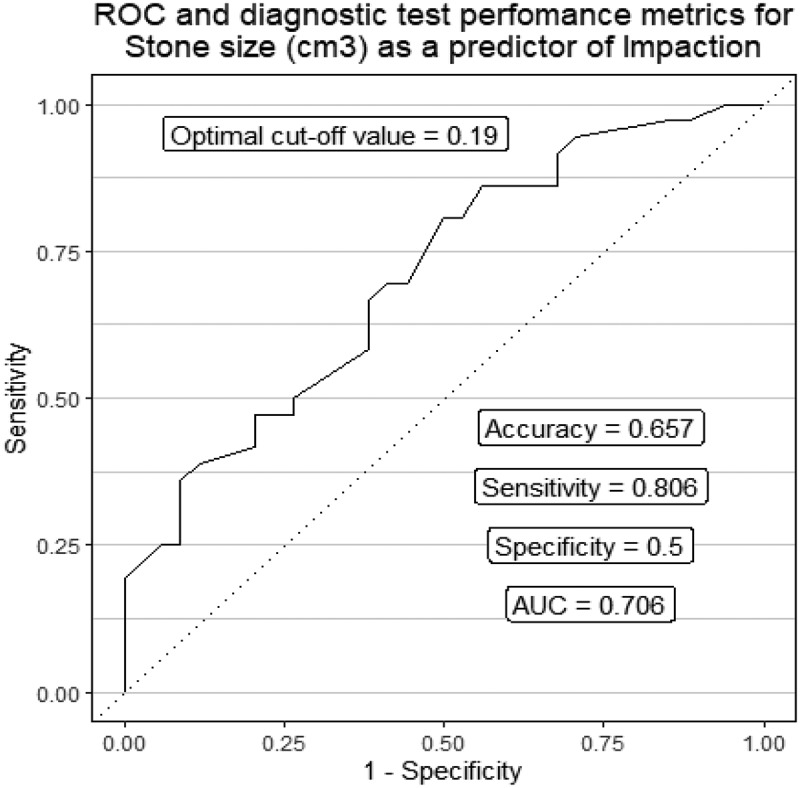
Diagnostic test performance of stone size as a predictor of impaction.

Stones with a volume of 0.19 cm3 or larger can be utilized as a prediction tool for impaction with high level of sensitivity (80.6%) and acceptable specificity (50%) (Figure 3).

## Discussion

Ureteric stone impaction can be confirmed during URS, where the stone is noticed to be adherent to the ureteral wall [13]. The choice of the optimal intervention for these stones remains a point of controversy, and no approved guidelines for their best management yet [14]. The advent of minimally invasive approaches has shifted the management of impacted stones away from open surgery [15].

The available armamentarium includes URS, extracorporeal shockwave lithotripsy (SWL), antegrade percutaneous nephrolithotomy (PCNL) and rarely laparoscopic ureterolithotomy [16]. Both URS and SWL represent the most used interventions with more efficacy and fewer complications than the others [17]. When URS is used in the management of impacted stones, it can be complicated with ureteral bleeding, injury, and subsequent stricture [9].

Our study showed no difference when comparing both groups regarding gender. The same observation was noted with Abdrabuh et al. [1]. This is contrary to what Ozbir et al. have found, where they found that impaction had a male predilection [12]. Another study by Samir et al. showed a higher stone passage rate in females [18]. Also, no difference in age was found between the 2 groups. This was agreed upon by Abdrabuh et al., Sarica et al. and Ozbir et al. [1,2,12]. While Elibol et al. found an increased incidence of stone impaction with advancing age [19].

Stone location has shown interesting findings. The upper ureteral location had a sensitivity of 65%, a specificity of 94% and a positive predictive value of 92% for prediction of impaction. Both Wang et al. and Ozbir et al. showed no site predilection for impaction [12,20].

In the present study, ureteral stone volume showed statistical significance between the 2 groups by univariate analysis. However, by multivariate analysis, it was proven to be a non-statistically significant predictor of impaction. Tran et al. had similar results, where they concluded that stone size and volume were able to predict impaction by univariate analysis. However, they failed to show such a relationship by multivariate analysis [8].

It has been long thought that impaction arises from the embedding of the ureteric stone into the mucosa leading to edema formation, and thus it has been postulated that UWT could be an indicator of this phenomenon. Indeed, our study, as well as those by Yoshida et al. and Sarica et al. proved this. [2,10]. We found that UWT cutoff value of 1.9 mm is an excellent predictor of impaction with 72.2% sensitivity, 94.1% specificity, and an overall accuracy level of 82.9%.

The increased ratio of HU of ureter below to above the stone was explained by Tran et al., Ozbir et al., and Deguchi et al. by the fact that HU of the ureter below the stone is having the HU like tissue density because of non-passage of urine beyond the stone [8,11,12]. However, our study showed a median ratio of 2.95 for the impacted group and 3.67 for the non-impacted group. The difference in ratio between the 2 groups was statistically non-significant.

In our study, hydronephrosis proved to be a reliable predictor for stone impaction. In agreement with this, Wang et al. and Ozbir et al. enrolled the degree of hydronephrosis into their ureteral stone impaction formula [12,20]. We failed to show any relation between stone density, perinephric fat stranding, CRP and ESR with impaction.

The limitations of this study are the small number of cases and the manual measurement of the different NCCT parameters. Also, many patients with intramural ureteric stones were excluded because of the failure to assess the HU of the ureter below the stone.

## Conclusion

Based on our results, UWT, marked hydronephrosis and the upper ureteric stone site are the reliable predictors for stone impaction. Subsequently, we can counsel patients about the proper management plan and avoid the side effects of prolonged MET using these parameters.
